# Thrombembolic Events in Hospitalized COVID-19 Patients: What is the Role of the Sex?

**DOI:** 10.1055/a-1585-9536

**Published:** 2021-08-12

**Authors:** Irit Nachtigall, Sven Hohenstein, Andreas Bollmann, Marzia Bonsignore, Daniela Husser, Ralf Kuhlen, Andreas Meier Hellmann

**Affiliations:** 1 Helios Kliniken Ost and Klinikum Emil-von-Behring, Berlin, Germany; 2 Heart Center Leipzig at University of Leipzig and Leipzig Heart Institute, Leipzig Germany; 3 Evangelische Kliniken Gelsenkirchen, Zentrum für Krankenhaushygiene und Infektiologie, Gelsenkirchen, Germany; 4 Helios Health, Berlin, Germany; 5 Helios Kliniken GmbH, Berlin, Germany

Over a year ago, the WHO declared COVID-19 a pandemic; from then on, all hopes were on the development of vaccines. So far, 4 vaccines have been approved in Europe. On March 11 ^th^ , 2021, the European Medicines Agency (EMA) reported ∼30 cases of thromboembolic events (TE) that were observed within 2 weeks after vaccinations with the AstraZeneca vaccine Vaxzevria, mostly being cerebral venous sinus thromboses in women younger than 60 years. Ca. 5 million people had received Vaxzevria in the EEA by then. Several European countries stopped their vaccinations with Vaxzevria temporarily.

COVID-19 infections increase the risk of developing TE. It has not yet been reported whether women develop more TE under Covid-19 than men. The aim of the present study was to determine the frequency, sex distribution and risk factors of TE among SARS-CoV-2 positive patients.

## Methods

We analyzed claims data from 83 hospitals in the Helios Group. All patient 19,501 cases admitted between February 1 ^st^ , 2020 and February 8 ^th^ , 2021 with the International Statistical Classification of Diseases and Related Health Problems (ICD-10) code U07.1 (= PCR-confirmed infection with SARS-CoV-2), were included.

The following ICD-10 codes were used as definitions: Thrombocytopenia: D69.5, D69.6, pulmonary embolism: I26, thrombosis: I80, I81, I82, sinus vein thrombosis: G08, I67.6, I63.6. Only cases which were completed in the hospital, were included for hospital mortality ( *n* = 8,533).

We used the R software for statistical programming (version 4.0.2) for all analyses. The multivariable analyses of TE and in-hospital mortality were analyzed via logistic regression with log link function. In these models, we used sex, age (as numerical variable), comorbidities, and the frailty risk score 1 as predictors; in the models for in-hospital mortality, TE was an additional predictor.

## Results

**Fig. 1:**
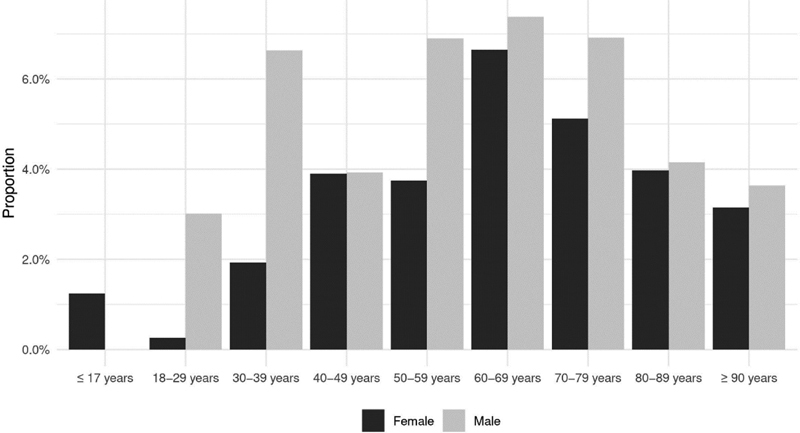
Proportion of patients with at least one thromboembolic event in Covid-19 inpatients by age and sex.

19,501 patients aged 0 - 103y (median 74y, Q25 = 59y, Q75 = 83y), 9,537 women (48.91%) and 9,964 men (51.09%) were included for the whole analysis, for calculation of the mortality 8,533 cases (85.64%) were included. Patient characteristis of the total cohort and the subcohort with thromboembolic events are shown in Table 1 . At least one TE was coded in 963 patients (4.94%) (433 pulmonary embolisms, 371 thrombocytopenias, 249 thromboses and 2 sinus vein thromboses, several events per patient being possible), incidence rate was 4,938 (per 100,000 cases; 95% CI: 4640–5254). TE occurred in 4.94% of all inpatients; men were affected by 5.73% (571 / 9.964) and women by 4.11% (392 / 9,537). The distribution of age and sex in thrmoboembolic events is shown in Figure 1 . In the multivariate regression analysis, independent risk factors for developing TE were among others male sex, lymphomas, liver diseases and congestive heart failure ( Table 2 ). TE were associated with an increased risk of death; the mortality rate was 20.7% in the group without TE and 39.8% in the group with such an event. (OR 2.28; 95% Cl 1.93–2.70).

**Table 1:** Patient characteristics of the total cohort and the subcohort of patients with thromboembolic events (TE)

Group	Total cohort	TE cohort
	Proportion ( *n* )	
**Sex**		
Male	51.1% (9,964)	59.3% (571)
Female	48.9% (9,537)	40.7% (392)
**Age**		
*Mean (SD)*	69.2 ± 18.7	69.9 ± 14.4
≤ 17 years	1.5% (290)	0.2% (2)
18 − 29 years	3.2% (616)	0.8% (8)
30 − 39 years	3.9% (762)	3.2% (31)
40 − 49 years	5.6% (1,099)	4.5% (43)
50 − 59 years	11.5% (2,238)	13.0% (125)
60 − 69 years	15.1% (2,952)	21.8% (210)
70 − 79 years	21.7% (4,229)	26.8% (258)
80 − 89 years	30.2% (5,894)	24.8% (239)
≥ 90 years	7.3% (1,421)	4.9% (47)
**Elixhauser comorbidity index**		
*Mean (SD)*	10.6 ± 11.2	21.3 ± 12.8
< 0	13.3% (2,586)	2.5% (24)
0	17.3% (3,365)	1.8% (17)
1–4	5.4% (1,050)	1.5% (14)
≥ 5	64.1% (12,500)	94.3% (908)
**Congestive heart failure**		
no	76.2% (14,863)	68.0% (655)
yes	23.8% (4,638)	32.0% (308)
**Cardiac arrhythmias**		
no	74.1% (14,443)	68.8% (663)
yes	25.9% (5,058)	31.2% (300)
**Valvular disease**		
no	92.6% (18,052)	89.5% (862)
yes	7.4% (1,449)	10.5% (101)
**Pulmonary circulation disorders**		
no	95.3% (18,594)	52.6% (507)
yes	4.7% (907)	47.4% (456)
**Peripheral vascular disorders**		
no	92.6% (18,054)	90.4% (871)
yes	7.4% (1,447)	9.6% (92)
**Hypertension, uncomplicated**		
no	56.2% (10,955)	59.4% (572)
yes	43.8% (8,546)	40.6% (391)
**Hypertension, complicated**		
no	88.5% (17,265)	85.9% (827)
yes	11.5% (2,236)	14.1% (136)
**Paralysis**		
no	95.1% (18,552)	95.2% (917)
yes	4.9% (949)	4.8% (46)
**Other neurological disorders**		
no	91.2% (17,785)	89.9% (866)
yes	8.8% (1,716)	10.1% (97)
**Chronic pulmonary disease**		
no	88.5% (17,267)	89.8% (865)
yes	11.5% (2,234)	10.2% (98)
**Diabetes, uncomplicated**		
no	83.2% (16,221)	80.9% (779)
yes	16.8% (3,280)	19.1% (184)
**Diabetes, complicated**		
no	88.4% (17,231)	88.7% (854)
yes	11.6% (2,270)	11.3% (109)
**Hypothyroidism**		
no	87.7% (17,109)	88.6% (853)
yes	12.3% (2,392)	11.4% (110)
**Renal failure**		
no	69.0% (13,460)	70.6% (680)
yes	31.0% (6,041)	29.4% (283)
**Liver disease**		
no	96.0% (18,725)	87.7% (845)
yes	4.0% (776)	12.3% (118)
**Peptic ulcer disease excluding bleeding**		
no	99.9% (19,487)	99.8% (961)
yes	0.1% (14)	0.2% (2)
**AIDS/HIV**		
no	100.0% (19,494)	99.9% (962)
yes	0.0% (7)	0.1% (1)
**Lymphoma**		
no	99.3% (19,358)	97.0% (934)
yes	0.7% (143)	3.0% (29)
**Metastatic cancer**		
no	97.7% (19,044)	95.2% (917)
yes	2.3% (457)	4.8% (46)
**Solid tumor without metastasis**		
no	95.1% (18,547)	92.3% (889)
yes	4.9% (954)	7.7% (74)
**Rheumatoid artritis/collaged vascular disease**		
no	98.2% (19,157)	97.6% (940)
yes	1.8% (344)	2.4% (23)
**Coagulopathy**		
no	95.5% (18,625)	57.7% (556)
yes	4.5% (876)	42.3% (407)
**Obesity**		
no	88.3% (17,216)	85.6% (824)
yes	11.7% (2,285)	14.4% (139)
**Weight loss**		
no	89.4% (17,436)	80.0% (770)
yes	10.6% (2,065)	20.0% (193)
**Fluid and electrolyte disorders**		
no	57.4% (11,198)	42.8% (412)
yes	42.6% (8,303)	57.2% (551)
**Blood loss anemia**		
no	99.5% (19,403)	98.5% (949)
yes	0.5% (98)	1.5% (14)
**Deficiency anemia**		
no	96.6% (18,845)	94.7% (912)
yes	3.4% (656)	5.3% (51)
**Alcohol abuse**		
no	98.1% (19,134)	96.2% (926)
yes	1.9% (367)	3.8% (37)
**Drug abuse**		
no	99.6% (19,430)	99.9% (962)
yes	0.4% (71)	0.1% (1)
**Psychoses**		
no	98.7% (19,256)	99.1% (954)
yes	1.3% (245)	0.9% (9)
**Depression**		
no	94.0% (18,334)	94.6% (911)
yes	6.0% (1,167)	5.4% (52)

**Table 2:** Results of multivariable analyses of thromboembolic complications

Variable	OR (95% CI)	*P* value
Age	1.00 (0.99 − 1.00)	0.10
Female sex	0.73 (0.63 − 0.83)	< 0.01
Frailty risk score	1.04 (1.02 − 1.05)	< 0.01
Congestive heart failure	1.40 (1.17 − 1.68)	< 0.01
Cardiac arrhythmias	1.06 (0.90 − 1.24)	0.51
Valvular disease	1.22 (0.96 − 1.54)	0.10
Peripheral vascular disorders	1.07 (0.84 − 1.36)	0.59
Hypertension, uncomplicated	0.81 (0.69 − 0.94)	< 0.01
Hypertension, complicated	0.87 (0.69 − 1.11)	0.26
Paralysis	0.67 (0.48 − 0.93)	0.02
Other neurological disorders	0.92 (0.73 − 1.16)	0.47
Chronic pulmonary disease	0.75 (0.60 − 0.93)	< 0.01
Diabetes, uncomplicated	1.05 (0.89 − 1.26)	0.55
Diabetes, complicated	0.85 (0.67 − 1.07)	0.18
Hypothyroidism	0.93 (0.75 − 1.15)	0.48
Renal failure	0.66 (0.56 − 0.78)	< 0.01
Liver disease	2.94 (2.33 − 3.70)	< 0.01
AIDS/HIV	2.36 (0.27 − 20.89)	0.44
Lymphoma	5.19 (3.36 − 8.00)	< 0.01
Metastatic cancer	1.65 (1.07 − 2.55)	0.02
Solid tumor without metastasis	1.22 (0.86 − 1.71)	0.26
Rheumatoid artritis/collaged vascular disease	1.43 (0.92 − 2.21)	0.11
Obesity	1.24 (1.02 − 1.51)	0.03
Weight loss	1.57 (1.32 − 1.88)	< 0.01
Fluid and electrolyte disorders	1.43 (1.23 − 1.67)	< 0.01
Deficiency anemia	1.27 (0.93 − 1.72)	0.13
Alcohol abuse	0.96 (0.65 − 1.41)	0.84

Quality of regression model tested with the Hosmer-Lemeshow test, indicating good calibration (χ ^2^ = 23.556; *p* = 0.428).

## Discussion

In our cohort of Covid-19 inpatients, TE occurred in approx. 5%; involved mostly pulmonary embolisms and affected mainly men in their 60ies. In addition to various pre-existing conditions, we found the male sex to be a major independent risk factor for the development of TE. TE are a common complication of COVID-19 and have been reported to occur in ca. 7% of inpatients treated with thromboembolism prophylaxis. 2 Several possible pathomechanisms have been discussed, including a direct endothelial damage 3 as well as an antibody-mediated activation of platelets via the Fcγ-IIa receptor. 4 Men are at an increased risk of a severe course of Covid-19 5 ; TE are presumably part of this multifactorial, gender-specific risk.

The antibody-mediated activation of platelets via the Fcγ-IIa receptor has also been suggested as pathomechanism for TE after Vaxzevria. 6 It is unclear, why younger women seem to be affected more often by this complication. In general, sinus vein thromboses occur mostly in younger women. 7 The EMA reviewed 62 cases of cerebral venous sinus and 24 of splanchnic vein thromboses reported until March 22th, 2021. Although most of the cases reported have occurred in women <60y, no specific risk factors like sex or age were confirmed; the risk for TE after the vaccinations was estimated at 1: 100 000.

In summary, we found that a large number of Covid-19 inpatients have thromboembolic complications, at a frequency that is 5000 times higher than the one of TE after Vaxzevria. Although similar pathomechanisms have been discussed for both, the development of TE in Covid-19 and after vaccination, these two phenomena differ clearly in the type of thromboses that occur and in the sex distribution. The observational design of our study based on claims data are a strong limitation; at best, the results can provide a signal, especially information on medications and their different impact on both sexes need to be addressed. To our opinion, the sex aspect of thromboembolic events in Covid-19 and after Vaxzevria has not been adequately addressed so far and needs further investigation.
